# Practical Recommendations for Navigating Digital Tools in Hospitals: Qualitative Interview Study

**DOI:** 10.2196/60031

**Published:** 2024-11-27

**Authors:** Marie Wosny, Livia Maria Strasser, Simone Kraehenmann, Janna Hastings

**Affiliations:** 1 School of Medicine University of St Gallen (HSG) St.Gallen Switzerland; 2 Institute for Implementation Science in Health Care University of Zurich (UZH) Zurich Switzerland; 3 Clinic for Internal Medicine, Family Medicine, and Emergency Medicine Kantonsspital St.Gallen (KSSG) St.Gallen Switzerland; 4 SIB Swiss Institute of Bioinformatics Lausanne Switzerland

**Keywords:** health care, hospital, information system, information technology, technology implementation, training, medical education, digital literacy, curriculum development, health care workforce development, mobile phone

## Abstract

**Background:**

The digitalization of health care organizations is an integral part of a clinician’s daily life, making it vital for health care professionals (HCPs) to understand and effectively use digital tools in hospital settings. However, clinicians often express a lack of preparedness for their digital work environments. Particularly, new clinical end users, encompassing medical and nursing students, seasoned professionals transitioning to new health care environments, and experienced practitioners encountering new health care technologies, face critically intense learning periods, often with a lack of adequate time for learning digital tools, resulting in difficulties in integrating and adopting these digital tools into clinical practice*.*

**Objective:**

This study aims to comprehensively collect advice from experienced HCPs in Switzerland to guide new clinical end users on how to initiate their engagement with health ITs within hospital settings.

**Methods:**

We conducted qualitative interviews with 52 HCPs across Switzerland, representing 24 medical specialties from 14 hospitals. The interviews were transcribed verbatim and analyzed through inductive thematic analysis. Codes were developed iteratively, and themes and aggregated dimensions were refined through collaborative discussions.

**Results:**

Ten themes emerged from the interview data, namely (1) digital tool understanding, (2) peer-based learning strategies, (3) experimental learning approaches, (4) knowledge exchange and support, (5) training approaches, (6) proactive innovation, (7) an adaptive technology mindset, (8) critical thinking approaches, (9) dealing with emotions, and (10) empathy and human factors. Consequently, we devised 10 recommendations with specific advice to new clinical end users on how to approach new health care technologies, encompassing the following: take time to get to know and understand the tools you are working with; proactively ask experienced colleagues; simply try it out and practice; know where to get help and information; take sufficient training; embrace curiosity and pursue innovation; maintain an open and adaptable mindset; keep thinking critically and use your knowledge base; overcome your fears, and never lose the human and patient focus.

**Conclusions:**

Our study emphasized the importance of comprehensive training and learning approaches for health care technologies based on the advice and recommendations of experienced HCPs based in Swiss hospitals. Moreover, these recommendations have implications for medical educators and clinical instructors, providing advice on effective methods to instruct and support new end users, enabling them to use novel technologies proficiently. Therefore, we advocate for new clinical end users, health care institutions and clinical instructors, academic institutions and medical educators, and regulatory bodies to prioritize effective training and cultivating technological readiness to optimize IT use in health care.

## Introduction

### Background

In contemporary medical education, students are considered digital natives, having grown up immersed in various digital technologies [1]. Despite this, the health ITs (HITs) used in clinical settings differ markedly from the digital systems that students are accustomed to in everyday life [2]. Concurrently, the practice of medicine is increasingly incorporating a wide range of digital technologies, making it vital for medical professionals to understand and effectively use specialist as well as administrative information systems in clinical settings [3]. Today’s digitally native generation is perceived to be adaptable and skilled with new technologies, often learning through exploratory methods such as “learning by doing” or “trial and error.” However, it has been reported that medical students often lack formal education in digital literacy or exposure to HITs [4-6]. In contrast, some countries have incorporated the instruction of digital tools and literacy into their university medical education curricula. For instance, the United States has introduced learning platforms targeting essential HIT skills and knowledge [7]. Furthermore, Switzerland has recently undergone medical curriculum revisions aimed at achieving a structured and accountable acquisition of fundamental knowledge and skills in digital technologies through compulsory coursework for all students, alongside optional specialization opportunities, while emphasizing the constraints of digitalization within the medical field [8].

Among medical educators, there is a consensus on the necessity for proficiency in electronic health record (EHR) use as well as clinical informatics competencies to ensure students’ success after medical school [9-11]. Furthermore, familiarity with IT systems has been linked to perceived ease of use and usefulness, ultimately leading to improved work-related performance [12]. This phenomenon, termed “technology readiness,” refers to an individual’s ability to embrace new technology and is of particular significance in health care [13]. Furthermore, medical students face the challenge of navigating a variety of digital tools across different hospital environments, complicating the seamless integration of these technologies into clinical workflows [14] and necessitating individually tailored training programs to ensure efficient operation across diverse systems [15]. However, medical education is inherently complex and requires the involvement of multiple stakeholders, including the students themself, educators and clinical faculty, academic institutions, health care institutions, developers, and government and regulatory bodies Although each of these groups is interdependent, they might nevertheless have different priorities [16-18]. Therefore, educators and academic and health institutes must continue to play a central role in developing evidence-based curricula, integrating cutting-edge technologies, and fostering innovative programs that align with these diverse needs [19]. Moreover, developers must collaborate closely with clinicians during product development to ensure innovations meet user needs and maintain safety and clinical relevance across health care settings [20].

In addition to students, experienced health care professionals (HCPs) transitioning to new hospital environments face requirements to rapidly learn new technologies and often face suboptimal or generic training programs at their onset, and in some cases, they may lack formal training in the relevant health information systems [21]. HCPs may undergo various transitions in the course of their medical careers, such as altering their practice focus, assuming new roles and responsibilities, relocating geographically, or reentering the workforce after a leave of absence [22]. These transitions are critically intense learning periods and entering new health care environments introduces individuals to new workflows, processes, technologies, and subsequent challenges in their adoption [22]. These are often caused by diverse and fragmented IT systems across different hospitals and departments, which may apply distinct approaches to use HIT [23,24]. Moreover, it has been demonstrated that HCPs often experience feelings of uncertainty due to substantial changes in workflows and care processes, and participation in comprehensive training programs, such as simulation-based training, significantly enhances their readiness to work in new environments [25,26]. Furthermore, adequate training has been associated with increased satisfaction and efficiency among HCPs using HIT [27,28], emphasizing the importance of well-planned training programs [29]. Moreover, proficiency in handling HIT not only enhances familiarity but also contributes to their seamless integration into efficient workflows, which is markedly improved when supported by structured training [30].

Despite these findings, the availability of training and skill programs often falls short of meeting the needs of HCPs [31]. The research underscores a deficiency in hospital training for new technologies, leading to challenges such as resistance to change [32]. This deficiency often stems from several factors, including limited time availability, infrequent provision of training sessions, and an overreliance on e-learning as the primary mode of training delivery within clinical settings [33]. Moreover, while staff training is acknowledged as crucial during HIT implementation, insufficient emphasis on postimplementation training for day-to-day activities persists [28]. In addition, recognizing the human and social aspects alongside technological considerations is key to successful HIT adoption [34]. To optimize training, programs should align with clinical practice and cater to individual learning needs [34].

In addition to the training challenges, users often face considerable challenges due to the technologies themselves, despite their promising advancements [35]. For example, the emerging opportunities presented by generative artificial intelligence (AI) and large language models require careful management and understanding to be fully used and realize their maximum potential [36,37]. Other major hurdles include outdated tools, the continuous implementation of new systems, and respective updates [38]; an increase in computer-centric work due to administrative tasks; and the growing volume of medical data in recent years [39]. Therefore, the effective use and unlocking of the full potential of HITs rely on the willingness and proficiency of HCPs to use them [28].

These challenges can be addressed by adhering to best practices in education and training [31]; however, the integration of HITs into daily routines can still be overwhelming for both aspiring and experienced HCPs entering new hospital environments or engaging with novel technologies for the first time. This can be particularly disadvantageous for medical students, leading to a suboptimal commencement of their careers marked by issues, such as reduced productivity, inefficiencies, increased costs, and significant frustration, and may contribute to work-related struggles with mental health [40,41]. Similarly, the dissatisfaction of experienced professionals working with digital tools based on their deficiency in general digital knowledge and a lack of formal basic digital training or education can lead to frustration and can contribute to emotional exhaustion and burnout [42,43]. In a previous study, we observed that a significant proportion of hospital-based HCPs feel frustrated by the use of HITs, affecting their well-being and leading to work-life balance challenges, increased stress levels, cognitive overload, and mental burden [44].

### Objectives of This Study

The goal of HIT design and implementation should be for HCPs to perceive IT as a blessing rather than a burden. To mitigate these challenges, it is important to offer thorough training on the core functionalities of systems to end users [45,46]. Furthermore, incorporating new digital skills into the future curricula of medical faculties and integrating them into continuous professional development is essential to ensure that HCPs stay up-to-date with digital innovations [47]. Therefore, the objective of this qualitative research study is to synthesize recommendations from HCPs aimed at offering actionable guidance on the most effective methods for seamlessly integrating new clinical end users, such as medical students and transitioning experienced professionals, into novel digital hospital environments. This includes enabling them to adeptly navigate HIT systems with ease while also informing other key stakeholders, such as educators, administrators, and developers, on strategies to enhance user satisfaction, work efficiency, and the overall effectiveness of health care delivery.

## Methods

### Study Design

The study was designed in accordance with the COREQ (Consolidated Criteria for Reporting Qualitative Research) checklist [48] (Multimedia Appendix 1). A qualitative interview study was conducted to investigate the personal experience of HCPs using digital tools in Swiss hospitals. While their overall experiences and a detailed description of methods are reported in a previous study [44], in this study, we report the advice and recommendations of HCPs for new clinical end users using digital tools in hospitals upon the start of their careers, transitioning to a new health care setting, or encountering new health care technologies.

### Participant Recruitment and Selection Process

HCPs with ≥6 months of experience with digital tools in the hospital were recruited for interviews in their native language, that is, German or English, excluding students and nonhospital-based HCPs. Participants were recruited using a combination of convenience sampling and strategic outreach through multiple channels, including web-based platforms, social media, clinic newsletters, hospital intranet systems, and printed flyers. To further increase diversity, snowball sampling was used alongside purposeful sampling to ensure medical discipline diversity. The objectives of the study were clearly communicated via the institution’s website, which included a dedicated sign-up form for interested individuals.

### Data Collection Process

Interviews were conducted either in person at various hospital sites in Switzerland or via video call, based on the participants’ availability and preferences, from May to August 2023, until data saturation occurred [49]. Following the collection of demographic background data as well as the experience of HCPs with digital technologies as reported elsewhere [44], we asked 2 questions aimed at receiving guidance and advice from HCPs on how new clinical end users can best use HIT in the hospital. We asked the study participants, “Based on your experience, what advice would you give to other healthcare professionals who are just starting to use the/digital tool/s?” and “In your opinion, what are some of the best practices for using digital tool/s effectively in a hospital setting?” The interviews were audio-recorded, transcribed using the software “Spoke” (Spoke Software, Inc [50]), reviewed for accuracy, and anonymized. Participants provided feedback on their transcripts, with revisions made accordingly. No response from study participants within one month was interpreted as acceptance of the transcript.

### Data Analysis

Concurrent data collection and analysis were performed using ATLAS.ti software (ATLAS.ti Scientific Software Development GmbH) [51]. An inductive thematic analysis approach, following the framework proposed by Braun and Clarke [52], was used to identify, analyze, and report patterns within the qualitative data, providing a flexible and robust method for gaining a rich, detailed, and nuanced understanding of participants’ experiences without the need for a predefined theoretical framework [52]. Furthermore, 2 authors (MW and LMS) independently immersed themselves in the interview data through repeated readings of the transcripts and performed inductive coding to directly derive themes from the raw data. Both authors independently generated a list of codes and subsequently engaged in iterative discussions to compare and harmonize their findings. Through these discussions, the authors refined and expanded the codes into a single codebook. To deepen the analysis, the authors took detailed notes to capture key data items, patterns, and relationships between different parts of the dataset. The fourth author (JH) was involved in data analysis to ensure comprehensive data coverage and provide an additional layer of validation. All 3 of these authors collectively grouped initial codes into broader themes, particularly focusing on recommendations for clinical end users in hospital settings. As part of the thematic analysis, themes were further examined and restructured to ensure they were comprehensive and coherent. The third author (SK) was involved to ensure the credibility of the findings and feasibility of the recommendations within a clinical context from the perspective of a person with a background in medical education. Narrative descriptions and clear definitions were crafted for each theme, and all quotes were translated into English for reporting. Finally, the findings were discussed with study participants to validate and ensure trustworthiness.

The data have been presented through illustrative quotes, which were carefully selected to represent the arguments presented in the interviews and do justice to the variety of perspectives shown within them. In the selection, we considered whether the quotes could be understood without the context in which they were originally reported.

### Ethical Considerations

This study was deemed exempt from formal review and approval by the Ethics Committee of the University of St. Gallen. All participants provided verbal consent immediately before each audio-recorded interview and were informed that their participation was entirely voluntary.

## Results

### Characteristics of Study Participants

We conducted interviews with 52 HCPs representing 24 different medical specialties across 14 hospitals in Switzerland, with an equitable distribution of gender and an average age of 40 (SD 10.18) years. Physicians constituted the predominant cohort, comprising 71% (37/52) of the sample, with 42% (22/52) occupying senior roles and 29% (15/52) categorized as resident physicians. Registered nurses constituted 29% (15/52) of the participants, with 19% (10/52) holding senior positions and 10% (5/52) functioning as regular staff nurses. Most HCPs reported using digital tools since the start of their professional careers. Most (32/52, 62%) HCPs reported having extensive experience with digital technologies that is exceeding a decade, while 13% (7/52) of the HCPs reported having an experience between 5 and 10 years, 19% (10/52) having an experience between 1 and 5 years, and only a small proportion of 6% (3/52) having an experience with digital tools between 6 months and 1 year (Table 1). The study participants reported using a comprehensive range of >100 digital tools, encompassing both hardware and software, designed to meet the medical and administrative needs of the hospital. The most commonly mentioned medical tools included the clinical information system, cited by 100% (52/52) of HCPs, followed by dictation and speech recognition software used by 42% (22/52) of the participants. Laboratory information systems were reported by 38% (20/52) of the participants, while knowledge databases were mentioned by 31% (16/52) of the participants, alongside picture archiving and communication systems, which were also reported by 31% (16/52) of the participants. Other commonly used tools included literature databases, reported by 15% (8/52) of the participants, remote patient monitoring tools reported by 15% (8/52) of the participants, and surgery planning tools reported by 15% (8/52) of the participants. On the administrative side, the most frequently mentioned digital tools were email programs, used by 63% (33/52) of the participants, followed by the MS Office 365 Suite (Microsoft Corporation), reported by 44% (23/52) of HCPs. Videoconferencing tools were mentioned by 31% (16/52), and shift scheduling software was mentioned by 23% (12/52) of the participants.

**Table 1:** Sociodemographic and professional characteristics of study participants (N=52).

Characteristics		Participants
**Hospital size, n (%)**		
	Large (>700 beds and >7000 staff)	11 (21)
	Medium (500-700 beds and 3000-7000 staff)	32 (62)
	Small (<500 beds and <3000 staff)	9 (17)
**Role, n (%)**		
	Senior physician	22 (42)
	Resident physician	15 (29)
	Senior registered nurse	10 (19)
	Staff registered nurse	5 (10)
**Sex, n (%)**		
	Female	25 (48)
	Male	27 (52)
Age (y), mean (SD)		40.31 (10.18)
**Age group (y), n (%)**		
	20-29	10 (19)
	30-39	17 (33)
	40-49	14 (27)
	50-59	9 (17)
	≥60	2 (4)
**Experience with digital tools (y), n (%)**		
	0.5-1	3 (6)
	>1-5	10 (19)
	>5-10	7 (13)
	>10	32 (62)
**Employment status (%), n (%)**		
	100	39 (75)
	80-90	10 (19)
	<80	3 (6)
**Medical discipline, n (%)**		
	Internal medicine, such as cardiology, endocrinology and diabetology, gastroenterology, geriatrics, hepatology, nephrology, and pneumology	19 (37)
	Surgical specialties, such as gynecology, neurosurgery, orthopedics, otorhinolaryngology, and urology	12 (23)
	Emergency and intensive care	8 (15)
	Cancer care, such as oncology and hematology, and radiation oncology	5 (10)
	Transdisciplinary	3 (6)
	Specialties, such as infectious diseases, ophthalmology, and psychiatry	3 (6)
	Diagnostics and imaging, such as pathology and radiology	2 (4)

### Ten Practical Recommendations for Clinical End Users

#### Principal Findings

**Figure 1:**
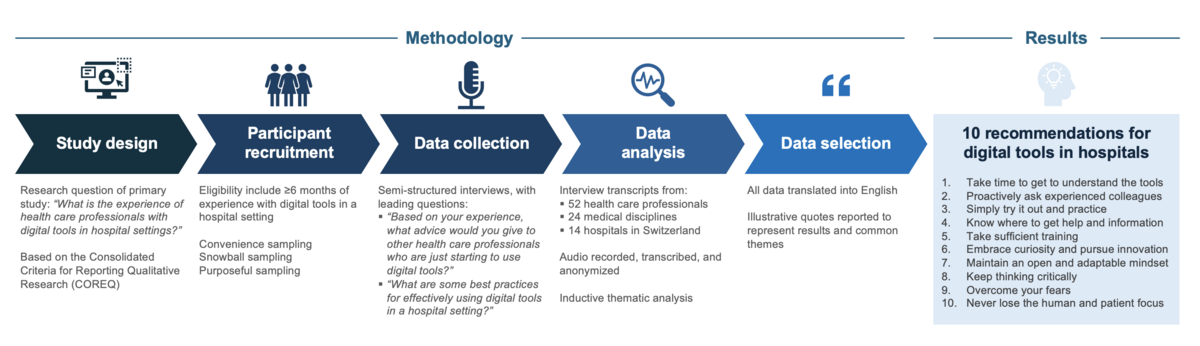
Study design, methodology, and ten recommendations for end users to navigate digital tools in hospitals.

We identified 187 quote annotations on advice and recommendations with 79 unique primary codes from all 52 interviews with HCPs. In total, 10 themes regarding the adaptation to digital tools for new end users emerged from the data related to technological aspects such as digital tool understanding (n=47); learning-related factors, including peer-based learning strategies (n=28), experimental learning approaches (n=16), knowledge exchange and support (n=14), and training approaches (n=12); and human-centric factors such as proactive innovation (n=33), an adaptive technology mindset (n=20), critical thinking approaches (n=19), dealing with emotions (n=16), and empathy in combination with human factors (n=12; Figure 1; Table 2). On the basis of these emerging themes, we synthesized 10 recommendations with subsequent specific actions to take for new clinical end users. These recommendations are aimed at medical and nursing students, experienced professionals who transition to new hospitals, as well as users encountering novel health care technologies. The coding tree illustrating the progression from raw data to final themes is depicted in Multimedia Appendix 2.

**Table 2:** Emerging themes and recommendations for using digital tools in the hospital.

Emerging themes (number of annotations)			Recommendations for new clinical end users		Specific actions to take
**Technology-related factors**					
	Digital tool understanding (47)	Take time to get to know and understand the tools you are working with		Thoroughly familiarize yourself with health information systems from the start and invest time to understand their functions and purpose for efficient outcomes. Use the given tools proactively, recognize their strengths and limitations, and prioritize data privacy and compliance with regulations.	
**Learning-based factors**					
	Peer-based learning strategies (28)	Proactively ask more experienced colleagues		Actively seek guidance from experienced colleagues and request tailored advice on navigating daily tasks particularly to adapt to the hospital settings and conditions.	
	Experimental learning approach (16)	Simply try it out and practice		Actively explore the diverse range of health information technologies and “give them a try” while adopting a “learning by doing” approach, and there is a minimal risk of system breakage through interactive exploration in a training setting. Remember that proficiency grows with consistent use and practical application.	
	Knowledge exchange and support (14)	Know where to get help and information		Take the initiative to familiarize yourself with where and how to seek assistance to know what to do when in need. Diligently document your digital tool learnings to enhance your proficiency in tool navigation, ensuring you have a resource to revisit for reference or refreshers.	
	Training approaches (12)	Take sufficient training		Demand targeted, comprehensive training tailored to your discipline-specific needs, ensuring you acquire essential skills and maximize effective use of health information technology while also advocating for sufficient time to be allocated for this training.	
**Human-centric factors**					
	Proactive innovation (33)	Embrace curiosity and pursue innovation		Actively try out new digital tools, functions, or approaches and advocate for digitization by challenging conventional perspectives. Moreover, acquire useful skills such as fast typing and text block preparation to elevate workflow proficiency.	
	Adaptive technology mindset (20)	Maintain an open and adaptable mindset		Embrace an open-minded approach to health information technologies and remain curious about emerging solutions, recognizing that change is constant and new solutions and technologies will continually emerge, requiring adaptability and a willingness to explore innovative alternatives.	
	Critical thinking approaches (19)	Keep thinking critically, and use your knowledge base		Maintain responsibility by critically thinking and interpreting tool decisions, questioning the “why” behind clinical workflows and remembering that tools support, rather than replace your actions.	
	Dealing with emotions (16)	Overcome your fears		Try to reflect on the specific causes of your anxiety and recognize that skill gaps or insecurities can be addressed through training. Advocate for maintaining a positive mindset toward health information technologies and embrace challenges with optimism, recognizing the diverse nature of hospital environments and situations.	
	Empathy and human factors (14)	Never lose the human and patient focus		Prioritize maintaining a strong human and patient focus through meaningful conversations and personal connections while integrating technical skills seamlessly into your routines, ensuring a balance between digital tools and the human element of patient care.	

#### Digital Tool Understanding

To take the time to thoroughly familiarize oneself with health information systems that are part of the daily work routines, including exploring all their functions, features, and possibilities, was highlighted by a large proportion of respondents (17/52, 33%), ideally right at the onset of hospital work (4/52, 8%). Although the importance of effective training was recognized, a lack of prioritization (11/52, 21%) and a shortage of dedicated time (6/52, 12%) were frequently mentioned. Investing time upfront, coupled with a profound comprehension of both the tool and its purpose, was emphasized to lay the groundwork for achieving satisfactory outcomes and operational efficiency (5/52, 10%). Moreover, proactive use of available tools and features was highlighted, as this contributes significantly to enhanced productivity (4/52, 8%) as well as emphasizing the inherent benefits of digital tools as a motivational factor for efficient use (3/52, 6%). Recognizing both the strengths and limitations of health information systems was reported as a critical aspect of informed tool use (4/52, 8%), along with asking the “why” before using a tool to ensure thoughtful evaluation (2/52, 4%). Furthermore, strict adherence and compliance to tool permissions to uphold operational integrity and regulatory standards were highlighted to be crucial, ensuring that tools are used within authorized boundaries (2/52, 4%). Moreover, a collective commitment to taking data privacy seriously was underscored (2/52, 4%), reinforcing a holistic approach to responsible and effective tool use:

It is crucial to dedicate sufficient time in the beginning to fully comprehend all the functions. We have numerous tools at our disposal, with predefined curves for various clinical scenarios. This allows you to simply check the appropriate boxes or remove them, saving you a considerable amount of time.

Resident physician, surgery

Take a moment to sit down and explore the system. Whenever you have the opportunity and are not occupied take some time to familiarize yourself with the system. If there is something you do not understand, do not hesitate to seek clarification.

Staff registered nurse, emergency medicine

I always prioritize investing time in understanding a new tool, to practice with it, and exploring all its possible features. This way, I can fully maximize its utility.

Senior physician, neurosurgery

#### Peer-Based Learning Strategies

One of the main advice points from HCPs was to proactively seek guidance from experienced colleagues (19/52, 37%). This also involved close observations of more senior HCPs in their interaction with tools (6/52, 12%) and engaging in social exchanges beyond clinical duties (4/52, 8%). Participants advised new end users not to hesitate but to actively approach and seek advice from their experienced colleagues, as this is considered essential for their professional development:

At times, the students may appear a bit reserved, and it might feel like they are wondering, “Is it permissible to inquire?” Absolutely, you are carrying out an essential task. Understanding the system and its operation is vital, and as a result, asking questions is important.

Senior physician, urology

Moreover, in the case of any queries, do not hesitate to seek guidance from your colleagues and adapt to the specific local conditions.

Resident physician, radiation oncology

Consequently, I believe the best advice is to approach an experienced resident physician who can precisely demonstrate what you need for your everyday life and what you can do without.

Resident physician, neurosurgery

#### Experimental Learning Approaches

The importance of actively engaging in the practice of digital tools was emphasized by a substantial number of participants (9/52, 17%), encouraging new end users to adopt a mindset of experimentation and urging them to “give it a try” and maintain a willingness to explore. Within this exploration, the study participants highlighted the potential discovery of hidden shortcuts and efficient methods. Another notable recommendation was the emphasis on the “learning by doing” approach (5/52, 10%). This approach advocates a mindset of trial and error, suggesting that new end users should engage in hands-on exploration by extensively testing the function of digital tools and navigating without confirming final actions. Moreover, HCPs also underscored the necessity of hands-on experience for skill enhancement (3/52, 6%), emphasizing that proficiency with digital tools is best cultivated through regular use and practical application. Furthermore, the assurance that new end users will not disrupt HITs in a training setting was reported (2/52, 4%), aiming to instill confidence in new end users as well as emphasizing that the exploration process often leads to the discovery of novel ways and functions to improve workflows:

Give it a try. Always be willing to explore, even if it doesn’t always lead to the simplest route. Many shortcuts and efficient methods are hidden within. It is a matter of trial and error, just click around a bit. There is no need to worry about breaking anything, in fact, you will often discover new ways to streamline and enhance your work.

Staff registered nurse, transdisciplinary

My advice to them would be to navigate and explore without necessarily confirming actions. Instead, take the time to review before finalizing decisions.

Staff registered nurse, emergency medicine

Ultimately, practice is key. Regularly working with computer programs naturally improves your proficiency. When it comes to diagnostic tools, simply looking at them will not make you better. You have to use them on patients to improve your skills.

Resident physician, intensive care

#### Knowledge Exchange and Support

Study participants highlighted the importance of knowing where to seek assistance and access information when required for digital tool navigation (7/52, 13%), which is an important aspect of self-empowerment in the early stages of a career. Furthermore, the management of one’s data, knowledge, as well as self-management was reported to be not merely a technical skill but a critical mindset (3/52, 6%). Participants stressed that using tools strategically and efficiently can serve as catalysts for improvement and personal growth, involving the active pursuit of solutions and the continuous expansion of technical skills. In the rather hectic work environment in the hospital, study participants recommended documenting learnings and insights, such as tool functions, processes, clicks, and shortcuts (3/52, 6%), which is not about creating a checklist but rather curating a personalized compendium of techniques to aid in navigating digital tools and their corresponding clinical workflows:

In our setting, we also provide numerous knowledge exchanges and knowledge-sharing sessions. Depending on one’s role, individuals gain automatic access to these resources. They can use this information for self-training and personal growth.

Senior registered nurse, transdisciplinary

When adapting to a new digital tool, it’s valuable to possess certain skills. You should be able to explore and experiment with the tool independently to some extent or know where to seek assistance.

Senior physician, radiology

Having a theoretical understanding of all the necessary steps is advantageous. It enables you to reference these steps as needed. Essentially, it is about empowering yourself to find solutions independently.

Resident physician, intensive care

#### Training Approaches

To succeed with HITs, targeted and sufficient training for the acquisition of the right skills was mentioned to be highly important (11/52, 21%) and should be demanded, with an emphasis on more specific training for highly discipline-specific tools (5/52, 10%). While the significance of thorough and impactful training has been acknowledged, there was a recurring sentiment that training sessions often fall short in effectiveness, characterized by a perceived lack of specificity (12/52, 23%):

I believe that receiving comprehensive training is of high importance.

Resident physician, psychiatry

In cases where individuals commence work at a new hospital, they have to join the introductory training and receive thorough instructions. Ideally, the hospital or the relevant programs should offer introductory days to provide a logical and structured explanation.

Staff registered nurse, intensive care

I consider training to be a vital aspect of this process.

Senior registered nurse, oncology and hematology

#### Proactive Innovation

Cultivating curiosity and thinking outside the box (7/52, 13%) were highlighted with the importance of maintaining professional inquisitiveness and actively trying out new digital tools. Moreover, advocacy for digitization (2/52, 4%) was another emerging theme with participants suggesting new end users to actively challenge conventional perspectives, showcasing the possibilities of HIT, and questioning the status quo to bring about more efficient and innovative health care practices. Acquiring specific skills for enhanced proficiency with digital tools (2/52, 4%) was also emphasized, including fast typing skills and preparation as well as the use of text blocks:

Maintain your curiosity and think outside the box. I often advocate for professional inquisitiveness.

Senior physician, geriatrics

I believe it is great when someone tries out new tools. I definitely encourage that.

Senior physician, pathology

Advocate for digitalization and challenge conventional perspectives. Showcase the possibilities and question the status quo.

Senior physician, infectious diseases

#### Adaptive Technology Mindset

A considerable group of participants (9/52, 17%) emphasized the importance of maintaining an open mind toward HITs. This involves fostering a mindset that not only welcomes change but also embraces diversity and remains inherently curious about emerging solutions. In addition, a distinct call for flexibility, particularly in response to diverse situations and contexts (2/52, 4%) was made. As the current landscape of health care technology demands adaptability, being receptive to innovations and navigating the intricacies of various systems prevalent in modern hospitals is crucial. Each hospital operates uniquely, shaped by local needs and specific medical disciplines. Therefore, new end users need to embrace this diversity, recognizing that digital tools are not one-size-fits-all solutions. Furthermore, it was mentioned that staying open to alternative tools and systems is crucial. Continuously exploring new technologies allows for the possibility that something unexpected may emerge, challenging initial expectations or existing knowledge and providing opportunities for growth and improvement:

It is smart to remain open to the possibility of alternative tools or systems that might offer improved solutions. Continuously exploring new options is crucial.

Senior physician, cardiology

In my opinion, the most critical aspect is to maintain an open mindset. It is essential to be receptive to innovations and the various systems that are now commonplace in nearly every hospital, such as the hospital information system.

Senior registered nurse, internal medicine

It is important to keep an open perspective, acknowledging that something different may emerge, contrary to your initial expectations.

Senior physician, cardiology

#### Critical Thinking Approach

Interview participants highlighted the importance of maintaining a sharp critical thinking stance while relying on one’s medical knowledge base, including the ability to accurately assess tool-generated recommendations (5/52, 10%). A solid understanding of tool functionality and purpose was emphasized for their effective use. Moreover, understanding the “why” behind clinical workflows and processes is important and can contribute to efficiently managing digital tools and tasks. Another recurrent theme mentioned by participants is the need to continuously think critically (5/52, 10%) with a skeptical mindset, questioning the accuracy of information provided in evaluations, and avoiding blind trust. In addition, participants emphasized the critical need for recognizing priorities in navigating digital tools and managing workflows (5/52, 10%). This guidance encourages new end users to cultivate a heightened awareness of task prioritization and, specifically, students should recognize structures and workflows (3/52, 6%), entailing comprehending the organizational processes within which these digital tools are applied, contributing to a more comprehensive understanding of their functional integration. In addition, participants stressed the concept that tools are meant to support and not replace critical thinking (3/52, 6%). While tools are valuable aids, these should complement and enhance the decision-making process of HCPs:

Always approach things with a critical mindset, questioning the accuracy of the information provided in evaluations. Avoid blind trust.

Senior physician, pneumology

Understanding the “why” behind your actions is essential. The younger generation often exhibits a strong desire to comprehend the significance of their tasks. They want to know not only what to do but also why it’s vital. For instance, understanding why accurate performance recording matters, as it directly impacts our job percentages. Knowing the “why” is crucial, why we perform certain tasks and how they contribute.

Senior registered nurse, gastroenterology

I think it is quite important to have a solid understanding of the functionality of the tools you use, whether it is AI software or a document. Understanding the tool’s purpose is the key to using it effectively.

Senior physician, cardiology

#### Dealing With Emotions

A significant portion of participants (10/52, 19%) emphasized the importance of reflecting on one’s attitude toward frustration from digital tool use and fear of failure and mistakes. The consensus was to trust that problems are solvable, showcasing a collective belief in the efficacy of maintaining a positive mindset. Participants (5/52, 10%) underscored the significance of maintaining composure and overcoming frustrations, endorsed by reflecting on a practical and effective strategy for handling challenges in a professional context. Moreover, participants highlighted the importance of maintaining a positive and optimistic mindset, even in challenging situations to navigate hurdles (3/52, 6%). Furthermore, the pivotal significance of confidence and trust in one’s professional skills was underscored, highlighting that cultivating belief in one’s abilities constitutes a key element in constructing resilience when confronting challenges (2/52, 4%). Significantly, the importance of recognizing the diverse nature of hospital environments was emphasized (2/52, 4%):

I believe the primary piece of advice is not to be intimidated, even among individuals who have grown up with technology.

Senior physician, cardiology

For those who seem a bit afraid about it, we demonstrate how things appear and function, working to dispel their concerns. We let them know that we have robust support to assist them in the initial phases and that they can use these tools without any qualms.

Senior registered nurse, transdisciplinary

There is really no need to worry about technology at this point. I do not believe there is. You do not have to be a technology enthusiast to grasp or utilize it. It is within reach for everyone.

Resident physician, neurosurgery

#### Empathy and Human Factors

Participants advised new end users, particularly medical students, to maintain a strong human and patient focus throughout their practice (5/52, 10%) and emphasize the significance of spending time with patients, particularly during conversations. Furthermore, participants stressed the need to integrate technical skills into daily routines while never forgetting the human element. Regardless of the HITs used, both patients and physicians are fundamentally human. Therefore, participants encouraged new end users to blend technical proficiency with empathy (2/52, 4%). In addition, the study participants recommended engaging in patient interaction and examination without digital tools (2/52, 4%) to establish personal connections with patients:

Do not underestimate the time spent with patients, particularly the patient conversation. In many cases, the patient provides valuable insights into their diagnosis. It is important to remember that above all, there is a human being sitting in front of you.

Senior physician, cardiology

While it is essential to learn and integrate technical skills into your daily routine, do not forget the human element. Both on the patient’s side and the physician’s side, it is about humans.

Senior physician, gynecology

We consistently emphasize to our younger colleagues that the patient is critical. Medicine is not an administrative task, but it is a discipline that focuses on treating patients. It is crucial to spend time with patients in the physician’s office, rather than getting lost in the multitude of tools available, which can lead to neglecting the human aspect of patient care.

Senior physician, surgery

By integrating these recommendations into early career initiatives, curricula as well as outcomes of national catalogs of learning objectives, onboardings, reentry programs, system implementation initiatives, and instructional methodologies, both new end users and clinical instructors would be enabled to successfully navigate digital tools within hospital settings. This integration would facilitate the cultivation of essential practical skills, digital literacy, and a positive change mindset. Such a comprehensive approach not only guarantees a robust beginning for new end users in their professional journeys or when engaging with novel tools but also maximizes instructors’ time efficiency. Consequently, this would significantly contribute to the overall improvement of resource management and health care quality.

## Discussion

### Principal Findings

Despite the longstanding acknowledgment of the crucial role of HITs and the widespread understanding that providing HCPs with sufficient training is vital for seamlessly integrating them into their clinical workflows and ensuring their successful implementation, a significant gap remains in the availability of effective training modalities, support, and dedicated time.

The objective of this study was to collect essential guidance from HCPs to provide new end users in the hospital with effective strategies for navigating and using HITs. However, implementation, and hence the adoption of technologies, is a complex process, involving a variety of factors and multiple stakeholders [16]. Therefore, the responsibility cannot solely rest on the end users, as their capabilities are limited and the digital tool landscape varies greatly depending on the context of the organization they work in, leading to differences in implementation and use. Hence, it is important to emphasize that while the ability of end users to adapt to new digital technologies is crucial, our recommendations have inherent limitations. Health care institutions, academic organizations, and governmental regulatory bodies must support new end users by facilitating their adaptation to emerging technologies and enhancing teaching methods and curriculum design to ensure a more comprehensive and beneficial learning experience. Given the ongoing development of new HITs and continuous changes in the health care landscape, there is a continuous need for learning and adaptation. To enhance the current situation comprehensively and effectively, it is imperative to address challenges from all stakeholder perspectives, encompassing clinicians and new clinical end users, health care organizations, academic institutions, and regulatory authorities.

### End Users’ and Clinicians’ Roles in Adopting New Technologies

New end users have a responsibility to actively engage with and adapt to new technologies, ensuring they are used effectively and efficiently within their professional roles. Throughout this process, colleagues and experienced HCPs play a critical role in mentoring students and new or transitioning clinicians. To foster peer-based learning methodologies and allow new end users to actively ask experienced colleagues, clinicians and institutions need to foster a dynamic learning environment. Moreover, senior HCPs should not only encourage new end users to ask questions but also actively create opportunities for exchange and facilitate direct interaction in diverse contexts. Furthermore, scheduling regular feedback sessions is imperative for refining digital skills. Offering mentorships adds an invaluable layer of support, providing new end users with a guiding hand through the complexities of their medical journey with HIT [53]. Another important recommendation in this study highlighted the necessity for new clinical end users to know where and how to seek assistance when needed. To address this, clinicians could take proactive measures to promote and facilitate knowledge exchanges and sharing sessions as it is known that fostering clinicians’ sharing of knowledge regarding health technology is of high importance for the adoption and diffusion of HIT [54].

In addition, the significance of documenting learned processes and taking notes on user journeys as well as keyboard shortcuts has been emphasized by study participants, which also has been widely recognized by most educators who consider note-taking a critical component in learning [55]. However, note-taking practices have predominantly transitioned to digital devices and platforms, particularly within medical education contexts. Furthermore, disparities arise in the documentation habits of medical students, who heavily rely on mobile devices such as smartphones and iPads to record their learning experiences, which then declines in clinical settings, often due to resistance and ambivalence from educators and regulators regarding mobile device use in such sensitive contexts [56]. As a result, student-centered approaches to address issues related to digital note-taking and device portability must be explored in line with the development of ethically sustainable guidelines and a transparent code of conduct for the use of mobile note-taking devices during patient interactions [56].

Furthermore, senior HCPs and educators should recognize their responsibilities as role models. They should highlight the importance of maintaining a patient-centric approach amid vast digital data and additionally demonstrate the appropriate use of technology by sharing only trusted information, providing constructive feedback, adhering to norms and ethics, and fostering teamwork [57]. Role modeling is pivotal in shaping the professional development of medical trainees, and it is widely acknowledged in medical education as it is associated with numerous positive outcomes in learning and the cultivation of professional identity [58].

Moreover, emerging technologies in health care require new end users and, specifically, students to become critical thinkers, which can be enhanced by instructors through leveraging Socratic questioning and discussions, case studies, and real-life scenarios to emphasize the importance of continuous critical thinking [59,60].

Finally, the study underscored the importance of maintaining a patient-centric approach while using digital tools, a sentiment echoed by participants. While patient-centered care remains a cornerstone of medicine, our interviews revealed concerns that some HCPs, particularly students engaging with digital tools in clinical settings, may prioritize technology over patient interaction. To preserve the invaluable human touch amid the rise of technology, new clinical end users must always be aware that digital tools should be used to complement or enhance instead of replace core clinical skills [61].

### Health Care Organizations’ Role in Supporting Technological Integration

Health care institutions are key to providing clinical settings and resources that allow learners to adapt to new HITs and professional workflows. Health care institutions and clinical instructors need to allocate and compensate for dedicated time for learning, particularly at the start of employment. This is crucial because common barriers to adopting digital tools include the need for additional training, the time required to learn and use these tools, limited curricular time, and the need for more specific training to enhance technological skills [62-64]. Moreover, the concept of a “sandbox” as a safe space and testing environment for encountering health care technologies should be considered within educational settings [65]. Clinical educators should emphasize the value of experimentation, encouraging new end users to try out new tools with the learning-by-doing approach being a cornerstone, where new end users engage in practical experiences to enhance their comprehension and proficiency [66]. In addition, reassurance is also crucial, and instructors need to emphasize that trying out things will not lead to breakages or irreversible mistakes in a training setting. For example, research findings indicate that simulation-based training programs for EHR use not only enhance technical proficiency but also contribute to a richer educational experience, fostering a deeper comprehension of EHR-related communication and data management and leading to improved medical decision-making [67]. Furthermore, virtual reality, which is a technology that generates a simulated version of reality, has proven to be a valuable digital education tool to gain digital proficiency and ultimately increase technology acceptance [68,69].

Furthermore, to allow new end users to engage in training and learn from it, clinical instructors need to design and provide comprehensive training sessions for the effective use of digital tools. Consideration should be given to both current digital tools that are already implemented as well as emerging technologies such as generative AI [53-55]. Therefore, best practices in adult training, including factors such as optimal session duration and the use of formats such as interactive hands-on workshops and blended learning approaches, should be considered [70]. In addition, to empower new end users in conquering their technological apprehensions, clinical instructors must proactively address common skepticism, worry, anxiety, or fears of implementing technology to overcome “technophobia” [71]. Providing guidance on managing and overcoming these concerns is essential, with a focus on nurturing self-confidence through exposure strategies [71]. On top of that, experimental learning approaches were recommended to new end users; therefore, instructors should prioritize hands-on experience and guide practice sessions to enhance new end users’ proficiency in using HITs.

In addition, health care organizations need to provide at-the-elbow support by having knowledgeable personnel on-site to assist users in real time, particularly during the initial phases of EHR implementation [72]. This approach reduces disruptions and helps users adapt more efficiently. In addition, organizations should create self-help resources, such as wikis and on-screen assistance, to empower users with tools for independent troubleshooting, thereby improving long-term system adoption and operational efficiency. Moreover, instructors should foster a culture of open communication and establish a supportive and psychologically safe learning environment where new end users feel empowered to ask questions, propose ideas for improvement, or voice their concerns [73]. Furthermore, sharing success stories and real-world examples, along with having technology champions as promoters, are known to support its adoption and serve as powerful motivators for overcoming technological fears [74,75].

Besides that, to support an open-minded mindset among new end users, medical educators need to actively encourage new end users to embrace receptivity to new ideas, technologies, and approaches. Simultaneously, instructors should emphasize the significance of flexibility, encouraging new end users to adapt their methods and strategies based on the specific situations and contexts they encounter and also which they encounter during educational and curriculum change processes [76]. This principle also applies to fostering the abilities of new end users to think creatively outside the box and remain curious. Instructors play a pivotal role in encouraging new end users to actively explore innovative approaches and tools to enhance their daily workflows. Simultaneously, it is essential to underscore the importance of maintaining stringent data privacy standards while emphasizing data protection and security, which have been reported to often be underestimated in curricula and should be transparently taught according to clear guidelines and appropriate experts [77].

### Academic Institutions’ Role in Adopting Adequate Curricula

Academic institutions need to design up-to-date curricula that incorporate the latest technological advancements and innovative teaching approaches to better prepare students for modern health care challenges. Educators should be founded on a collaborative approach, understanding the needs of learners while aligning with the teaching methodologies and objectives set by the instructors [78]. Moreover, educators need to dedicate time to identifying the appropriate technologies to achieve the learning outcomes of their teaching program and must be willing to adapt their teaching practices [79]. Analysis of educational theories and evidence-based adult learning principles have already provided practical insights for clinical teachers seeking to enhance their effectiveness. Key implications include promoting active learning, retrieval of knowledge, and creating a psychologically safe environment [80]. Moreover, guiding learners in goal setting, fostering collaboration, and aligning teaching with authentic tasks contribute to a comprehensive educational approach [80].

At the same time, educators must have a deep understanding of the curriculum and recognize the significance of clearly defining learning objectives and curriculum goals of HIT, which is also essential for effectively assessing and providing summative feedback on student performance in terms of constructive alignment [81]. Furthermore, it is crucial to ensure these learning objectives are not only articulated but also standardized and reflected in formal documentation, such as the national competence-based catalog of learning objectives, serving as a foundational framework, guiding the development of suitable training courses and facilitating the implementation of assessments [82-84]. Moreover, academic institutions must not only use design thinking to redesign curricula in response to emerging challenges but, more importantly, integrate this approach into medical education itself, teaching HCPs how to apply it effectively in their own practice, as it has already been shown to enhance patient experiences, improve clinical outcomes, and refine medical training [85,86].

Moreover, it should be ensured that training materials are user-friendly, interactive, up-to-date, and easily accessible [87,88] and that there is sufficient and dedicated time allocated for training and flexible self-learning opportunities [89]. In addition, either in the e-learning or the in-person setting, a human-centered design approach for formal training rooted in implementation science, with a focus on participation engagement, should be encouraged [90,91]. In addition, a growth mindset should be cultivated, emphasizing that learning is an ongoing process, promoting openness to adapting to new tools and technologies in the future [92].

### Governmental and Regulatory Bodies’ Roles in Setting Standards and Reforms

Governments and regulatory authorities must establish policies and guidelines that ensure the integration of emerging technologies into medical education while maintaining safety and quality standards. For instance, in response to recent developments, governments must establish comprehensive policies that facilitate the integration of AI and large language models into health care systems while simultaneously addressing critical ethical concerns, such as data privacy, patient safety, and well-being [93]. Political influences in medical education, though often contentious, can be beneficial if they promote public safety and evidence-based pedagogy [94]. Regulatory bodies also have a responsibility to standardize curricula, ensure quality assurance, and provide funding for innovative teaching methods to better prepare medical students for technological advancements in health care [95]. Furthermore, physician training curricula and professional development programs should emphasize the significance of digital health competencies, encompassing data, knowledge, and self-management, and demonstrate how technology can be optimally leveraged along self-management strategies to enhance medical practice and ensure quality health care [96]. Through these actions, governments need to support to future-proof the health care system, ensuring that medical students and HCPs are equipped with the necessary skills and tools to navigate the ever-evolving clinical landscape.

### Limitations

Our study has notable strengths, but it also has limitations. Through the inclusion of a diverse range of study participants and adherence to best practices in qualitative research, we ensured credibility through participant engagement, researcher reflexivity, and transparent data collection and analysis. Nevertheless, the use of convenience sampling may have introduced a self-selection bias, potentially influencing the recommendations, attitudes, and expectations expressed by participants. Furthermore, because the data exclusively stem from interviews, there may be a deficiency in structured observations regarding HCPs’ interactions with digital tools. Moreover, while the absence of field notes may have limited our ability to capture nonverbal cues or contextual nuances that could have added further depth to the analysis, the detailed transcriptions and comprehensive audio recordings already ensured rich, in-depth data collection. Furthermore, it would be advantageous to include perspectives from new end users, such as medical students or experienced HCPs transitioning into new hospital settings or encountering novel technologies, as well as other stakeholders including academic institutions, developers, or regulatory bodies. Finally, the study’s location in Switzerland may have constrained its applicability to other health care systems, and the focus on hospital settings may have limited generalizability to the primary care context. Subsequent research could explore more discipline- or tool-specific insights as well as other contexts and health care environments. Future research could explore more discipline- or tool-specific insights, examining various contexts and health care environments beyond the scope of this study.

### Conclusions

In conclusion, we want to highlight the importance of comprehensive education and effective training strategies in facilitating the seamless integration of new end users into the IT landscape within hospital settings. Equally crucial is the proficiency of clinical instructors, who play a vital role in not only designing but also imparting these strategies to ensure a successful and efficient adoption process. The synergy between well-structured education and adept training is fundamental to harnessing the full potential of digital tools in health care environments.

New end users should actively seek guidance from their experienced colleagues, fostering collaboration among peers with critical support from instructors. It is crucial for them to invest time in understanding tools, emphasizing comprehensive functionality during dedicated training sessions. Moreover, sufficient training is essential, with educators responsible for designing effective training modalities. Overcoming fears is advised, and instructors should proactively support new end users in addressing and resolving concerns. Furthermore, maintaining an open-minded approach to new technologies is vital, with active engagement and experimentation being encouraged. Instructors should prioritize hands-on experience and guide practice sessions. New end users should sustain critical thinking, leveraging medical knowledge with real-life scenarios incorporated by instructors. In addition, awareness of where to seek help and information has been reported as highly important. Staying curious and thinking innovatively is emphasized and, importantly, new end users must not lose sight of the human and patient aspect when using digital tools, particularly in direct patient interaction.

The diligent adoption of these recommendations holds the potential to catalyze a substantial enhancement in the digital proficiency of new end users and resource management of their respective clinical instructors, thereby contributing significantly to the optimization of HCPs’ work experience and contributing to an overall elevation of health care quality.

## Appendix

Multimedia Appendix 1 Consolidated Criteria for Reporting Qualitative Research: the 32-item checklist.

Multimedia Appendix 2 Coding tree illustrating the development of themes through inductive thematic analysis.

## Abbreviations

AI: artificial intelligence
COREQ: Consolidated Criteria for Reporting Qualitative Research
EHR: electronic health record
HCP: health care professional
HIT: health IT

## Data Availability

Data generated and analyzed during this study are available from the corresponding author on reasonable request. Full interview transcripts and audio files are not publicly available due to data protection reasons and the privacy of the participants.
