# Supplementary material for: Practical Recommendations for Navigating Digital Tools in Hospitals: Qualitative Interview Study
Source: JMIR Med Educ. 2024 Nov 27;10:e60031. doi: 10.2196/60031 (PMC11635325; doi:10.2196/60031)
Supplement: Multimedia Appendix 1 [file mededu_v10i1e60031_app1.docx]

**Practical Recommendations for Navigating Digital Tools in Hospitals: Qualitative Interview Study**

Marie Wosny^1,2^, Livia Maria Strasser^1^, Simone Kraehenmann^1,3^, Janna Hastings^1,2,4^

^1^School of Medicine, University of St. Gallen (HSG), St Gallen, Switzerland

^2^Institute for Implementation Science in Health Care, University of Zurich (UZH), Zurich, Switzerland

^3^Clinic for Internal Medicine, Family Medicine, and Emergency Medicine, Kantonsspital St.Gallen (KSSG), St. Gallen, Switzerland

^4^ Swiss Institute of Bioinformatics (SIB), Switzerland

**Supplementary Appendix 1**

## Consolidated criteria for reporting qualitative studies (COREQ): 32-item checklist

Developed from Tong A, Sainsbury P, Craig J. Consolidated criteria for reporting qualitative research (COREQ): a 32-item checklist for interviews and focus groups. International Journal for Quality in Health Care. 2007. Volume 19, Number 6: pp. 349 – 357

| **No. Item** | **Guide questions/ description** | **Reporting** |
| --- | --- | --- |
| **Domain 1: Research team and reﬂexivity** | | |
| **Personal Characteristics** | | |
| 1. Interviewer/facilitator | Which author/s conducted the interview or focus group? | - Marie Wosny  - Janna Hastings |
| 2. Credentials | What were the researcher’s credentials?  *E.g., PhD, MD* | - Marie Wosny: M.Sc., B.Sc.  - Livia Strasser: B.Sc.  - Simone Kraehenmann: MD, M.Sc.  - Janna Hastings: Prof., Dr. rer. Nat., M.Sc. B.Sc. |
| 3. Occupation | What was their occupation at the time of the study? | - Marie Wosny: Research assistant  - Livia Strasser: Student research assistant  - Simone Kraehenmann: Senior physician and medical representative assurance of learning  - Janna Hastings: Professor |
| 4. Gender | Was the researcher male or female? | - Marie Wosny: Female  - Livia Strasser: Female  - Simone Kraehenmann: Female  - Janna Hastings: Female |
| 5. Experience and training | What experience or training did the researcher have? | MW, LS and JH have experience and have been trained to conduct qualitative research in the dimension of healthcare, SK has extensive experience in the field of medical education. |
| **Relationship with participants** | | |
| 6. Relationship established | Was a relationship established prior to study commencement? | The research team that conducted the interviews had no prior relationships with most of the participants before the interviews took place. The team had only encountered four healthcare professionals before the interviews, whether through university, a research workshop, or personal connections. As a result, both the participants and the interviewers should be considered unbiased. |
| 7. Participant knowledge of the interviewer | What did the participants know about the researcher? *E.g., personal goals, reasons for doing the research* | The objective of this study was communicated through the institute’s website and the interview sign-up form. Moreover, the objectives of the study and research purpose were reiterated at the outset of each individual interview. Interviewees were not informed of any personal objectives or interests unless participants specifically inquired. |
| 8. Interviewer characteristics | What characteristics were reported about the interviewer/ facilitator? *E.g., bias, assumptions, reasons, and interests in the research topic* | Information regarding the interviewers’ characteristics and their professional backgrounds was provided on the institute’s website. At the commencement of each interview, during the introduction phase, interviewees were informed of the interviewers’ educational backgrounds and current occupations (e.g., Marie Wosny’s expertise in molecular oncology and Janna Hasting’s specialization in bioinformatics and computer science). Interviewees were only made aware of other personal attributes if they directly inquired. |
| **Domain 2: Study design** | | |
| **Theoretical framework** | | |
| 9. Methodological orientation and Theory | What methodological orientation was stated to underpin the study? *E.g., grounded theory, discourse analysis, ethnography, phenomenology, content analysis* | The study was based on a qualitative research methodology conducting in-depth semi-structured interviews.  Inductive thematic analysis was performed (Braun V, Clarke V, 2006). |
| **Participant selection** | | |
| 10. Sampling | How were participants selected? *E.g., purposive, convenience, consecutive, snowball* | Participant recruitment and selection were carried out using a combination of diverse sampling approaches. Convenience sampling was employed, allowing eligible participants to register through an online form. Additionally, the snowballing technique was utilized to enhance diversity across demographics, clinical expertise, and experiences. Moreover, purposive sampling was conducted to encompass a wide spectrum of clinical areas of expertise and functions, ensuring a comprehensive perspective on the research topic. |
| 11. Method of approach | How were participants approached? *E.g., face-to-face, telephone, mail, email* | Following the participants’ sign-up and their agreement to take part in the study, the initial communication was established either through email or phone. Most of the semi-structured interviews were conducted in person, while a portion were facilitated via video calls, based on the availability of the study participants. |
| 12. Sample size | How many participants were in the study? | A total of 52 participants took part in the study. |
| 13. Non-participation | How many people refused to participate or dropped out? Reasons? | A total of six participants discontinued their involvement in the study. Reasons for dropping out included time constraints, reconsideration of suitability for the study, or being unreachable after initially agreeing to participate. |
| **Setting** | | |
| 14. Setting of data collection | Where was the data collected? *E.g., home, clinic, workplace* | The interviews were conducted in person at various locations, including hospital sites, nearby cafeterias/cafes, and the university, based on the participant’s preference. Additionally, some interviews were held through video calls. |
| 15. Presence of non-participants | Was anyone else present besides the participants and researchers? | No other individuals were present. |
| 16. Description of sample | What are the important characteristics of the sample? *E.g., demographic data, date* | During the interview, all participants were employed at hospitals in Switzerland and met the criteria for study participation. |
| **Data collection** | | |
| 17. Interview guide | Were questions, prompts, guides provided by the authors? Was it pilot tested? | The interview guideline was pilot-tested before the first interview. The participants were informed that no preparation was needed before the interview. The overall interview flow and agenda were explained during the personal introduction. |
| 18. Repeat interviews | Were repeat interviews carried out? If yes, how many? | None of the interviews was repeated. |
| 19. Audio/visual recording | Did the research use audio or visual recording to collect the data? | All interviews were recorded in audio format following the participants’ consent. |
| 20. Field notes | Were ﬁeld notes made during and/or after the interview or focus group? | No field notes were taken. |
| 21. Duration | What was the duration of the interviews or focus group? | The interviews had an average duration of 28 min. |
| 22. Data saturation | Was data saturation discussed? | The research teams consistently discussed data saturation. |
| 23. Transcripts returned | Were transcripts returned to participants for comment and/or correction? | Interview transcripts were provided to the participants for comment and corrections. Subsequent modifications were made based on their input and feedback. If no response or feedback to the transcript was received within a month, it was regarded as acceptable. |
| **Domain 3: analysis and ﬁndings** | | |
| **Data analysis** | | |
| 24. Number of data coders | How many data coders coded the data? | Data coding was performed by the MW and LS. |
| 25. Description of the coding tree | Did authors provide a description of the coding tree? | NA |
| 26. Derivation of themes | Were themes identiﬁed in advance or derived from the data? | Thematic analysis was employed to identify, analyze, and present first-order concepts, second-order themes, and aggregated dimensions that emerged from the data. These aspects portray the recommendations of healthcare professionals for new clinical end-users approaching new digital tools in hospital settings. |
| 27. Software | What software, if applicable, was used to manage the data? | For data analysis and management, “ATLAS.ti” software was used. |
| 28. Participant checking | Did participants provide feedback on the ﬁndings? | First-order concepts, second-order themes, and aggregated dimensions, along with emerging recommendations, were presented and discussed with study participants in an in-person meeting. |
| **Reporting** | | |
| 29. Quotations presented | Were participant quotations presented to illustrate the themes/ﬁndings? Was each quotation identiﬁed? *E.g., participant number* | Participant quotations were presented to illustrate themes and findings. Each quotation is attributed to the participant’s professional role, seniority, and medical discipline. |
| 30. Data and ﬁndings consistent | Was there consistency between the data presented and the ﬁndings? | Yes, there was consistency between the data presented and the findings, please refer to “Results”. |
| 31. Clarity of major themes | Were major themes clearly presented in the ﬁndings? | Yes, the ten merging themes were clearly presented in the findings, please refer to “Results”. Moreover, recommendations were discussed in the “Discussion” section. |
| 32. Clarity of minor themes | Is there a description of diverse cases or discussion of minor themes? | Yes, there is a description of diverse cases and a discussion of minor themes, please refer to “Results”. |
