# Supplementary material for: Practical Recommendations for Navigating Digital Tools in Hospitals: Qualitative Interview Study
Source: JMIR Med Educ. 2024 Nov 27;10:e60031. doi: 10.2196/60031 (PMC11635325; doi:10.2196/60031)

**Practical Recommendations for Navigating Digital Tools in Hospitals: Qualitative Interview Study**

Marie Wosny^1,2^, Livia Maria Strasser^1^, Simone Kraehenmann^1,3^, Janna Hastings^1,2,4^

^1^School of Medicine, University of St. Gallen (HSG), St Gallen, Switzerland

^2^Institute for Implementation Science in Health Care, University of Zurich (UZH), Zurich, Switzerland

^3^Clinic for Internal Medicine, Family Medicine, and Emergency Medicine, Kantonsspital St.Gallen (KSSG), St. Gallen, Switzerland

^4^ Swiss Institute of Bioinformatics (SIB), Switzerland

**Supplementary Appendix 2**

## Coding tree illustrating the development of themes through inductive thematic analysis


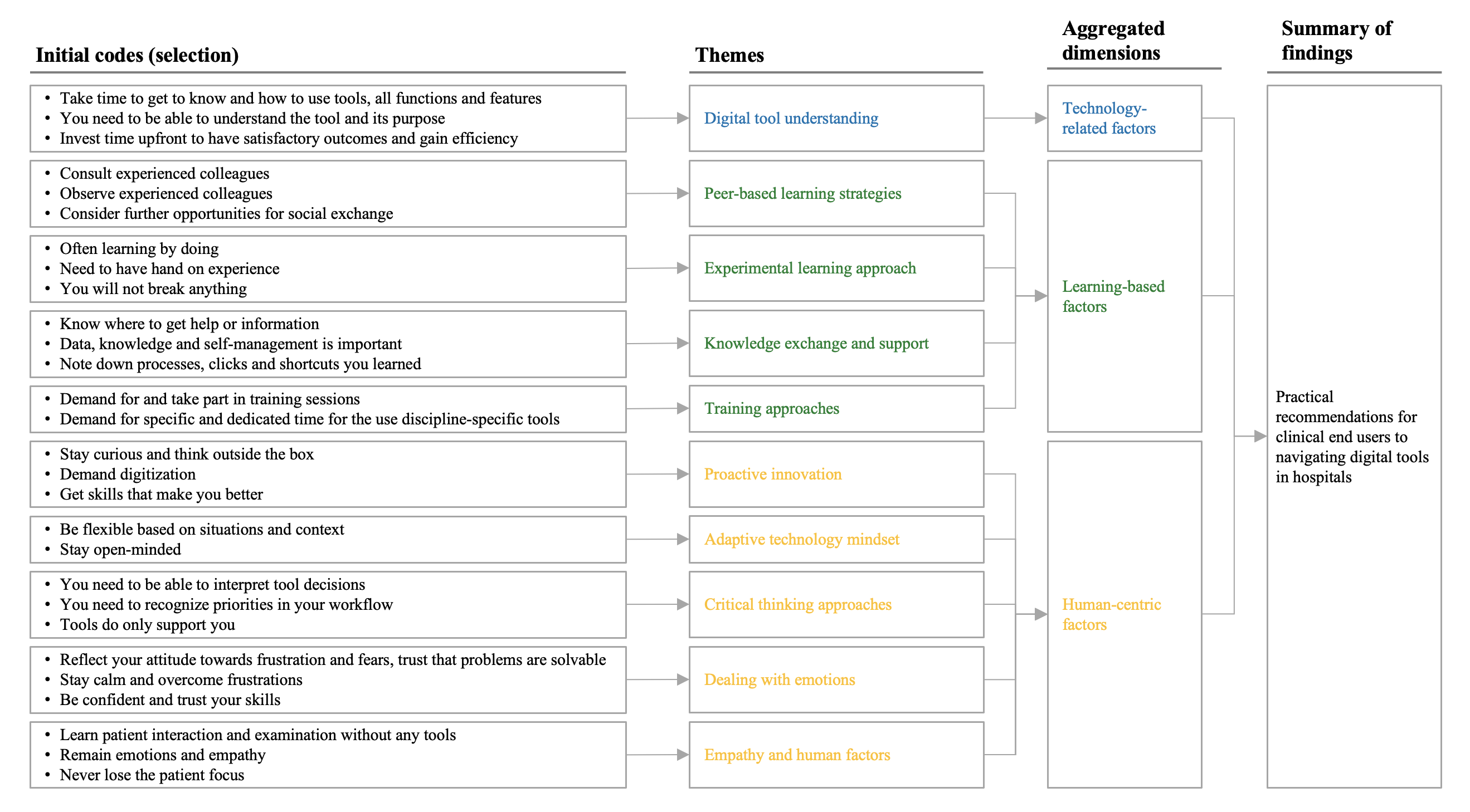

Supplement: Multimedia Appendix 2 [file mededu_v10i1e60031_app2.docx]
